# Clinical characteristics of patients with laboratory-confirmed influenza A(H1N1)pdm09 during the 2013/2014 and 2015/2016 clade 6B/6B.1/6B.2-predominant outbreaks

**DOI:** 10.1038/s41598-018-34077-4

**Published:** 2018-10-23

**Authors:** Yu-Chia Hsieh, Kuo-Chien Tsao, Ching-Tai Huang, Kuang-Yi Chang, Yhu-Chering Huang, Yu-Nong Gong

**Affiliations:** 1 Department of Pediatrics, Chang Gung Children’s Hospital, Chang Gung Memorial Hospital, Chang Gung University, College of Medicine, Taoyuan, Taiwan; 2 Department of Laboratory Medicine, Linkou Chang Gung Memorial Hospital, Taoyuan, Taiwan; 3 Research Center for Emerging Viral Infections, College of Medicine, Chang Gung University, Taoyuan, Taiwan; 4 Department of Medical Biotechnology and Laboratory Science, College of Medicine, Chang Gung University, Taoyuan, Taiwan; 5 Division of Infectious Diseases, Department of Medicine, Chang Gung Memorial Hospital, Chang Gung University, College of Medicine, Taoyuan, Taiwan; 6 Division of Biostatistics, Graduate Institute of Epidemiology and Preventive Medicine, National Taiwan University, Taipei, Taiwan; 7 Department of Anesthesiology, Taipei Veterans General Hospital and National Yang-Ming University School of Medicine, Taipei, Taiwan

## Abstract

A novel pandemic influenza A(H1N1)pdm09 virus emerged in 2009 globally, and it continues to circulate in humans. The National Influenza Surveillance Network in Taiwan identified five A(H1N1)pdm09-predominant seasons, representing the 2009/2010, 2010/2011, 2012/2013, 2013/2014, and 2015/2016 outbreaks from 2009 to 2016. Independently, a retrospective cohort study (which enrolled 639 infected patients during the five seasons) was conducted at Chang Gung Memorial Hospital to explore the risk factors associated with influenza A(H1N1)pdm09-related complications. A phylogenetic analysis of hemagglutinin (HA) sequences showed that the circulating A(H1N1)pdm09 virus belonged to clades 1, 2, and 8 in 2009/2010; clades 3, 4, 5, and 7 in 2010/2011; clades 7 and 6C in 2012/2013; clades 6B in 2013/2014; and 6B/6B.1/6B.2 in 2015/2016. Compared to individuals infected in non-6B/6B.1/6B.2 seasons (2009/2010, 2010/2011, and 2012/2013), those infected in 6B/6B.1/6B.2 seasons (2013/2014 and 2015/2016) were at higher risk for influenza-related complications (adjusted odds ratio [aOR]: 1.6, 95% confidence interval [CI]: 1.0–2.8), pneumonia (aOR: 1.78, 95% CI: 1.04–3.04), mechanical ventilation (aOR: 2.6, 95% CI: 1.2–5.6), and acute respiratory distress syndrome (aOR: 5.5, 95% CI: 1.9–15.9). For the increased severity of infection during the influenza A(H1N1)pdm09 clade 6B/6B.1/6B.2 seasons, aspects related to the antigenic change of A(H1N1)pdm09 virus, immune response of the host, and environmental factors required further investigation.

## Introduction

In June 2009, the World Health Organization (WHO) recognized a global human epidemiological event by announcing the detection of the first influenza pandemic of the 21st century that was caused by a novel influenza A(H1N1) virus^1^. This novel influenza A(H1N1)pdm09 virus continues to circulate (with antigenic evolution) in humans, and it contributes to normal seasonal epidemics of influenza^2^.

The 2013/2014 clade 6B-predominant season in the United States represented the first influenza A(H1N1)pdm09 season since the emergence of the virus in 2009, and it was characterized by elevated rates of hospitalization among adults aged 50–64 years^3,4^. In the 2015/2016 season, several European countries reported a high number of severe cases and outcomes in the groups at risk and healthy young adults; these events were associated with A(H1N1)pdm09 clade 6B.1 infection^5,6^. Since 1999, the Taiwan Centers for Disease Control (CDC) has established a nationwide surveillance system requiring contract virologic laboratories to perform continuous virologic surveillance for respiratory viruses, particularly influenza and enteroviruses; this system was established after an epidemic of enterovirus 71 in 1998^7^. The long-term National Influenza Surveillance Network described the epidemiologic pattern of circulating viruses, and it has successfully identified the outbreaks of severe acute respiratory syndrome (SARS)-associated coronavirus and adenovirus^8,9^. Moreover, it also identified the novel H7N9 and H6N1 influenza viruses^10^. Following the large magnitude of A(H1N1)pdm09 activity from July 2009 to January 2010, a second A(H1N1)pdm09 epidemic, evidenced by a sharp increase in the number of hospitalized patients and fatal cases, occurred in the winter of 2010^11^. Subsequently, A(H1N1)pdm09 has persisted at varying levels in the 2015/2016 season, when another A(H1N1)pdm09 epidemic occurred. The 2015/2016 outbreak included a sudden increase in the number of clinical cases with severe complications in adults aged 50–64 years.

Thus, the present study aimed to describe the A(H1N1)pdm09 epidemiological and virological data obtained from the National Influenza Surveillance Network that reported laboratory-confirmed influenza cases in intensive care units (a category 4 nationally notifiable disease) during a 7-year period extending from the 2009/2010 season to the 2015/2016 season. Independently, we conducted a retrospective cohort study at Chang-Gung Memorial Hospital to analyze the clinical characteristics and complications of patients with A(H1N1)pdm09 virus infection in the five A(H1N1)pdm09-predominant seasons (2009/2010, 2010/2011, 2012/2013, 2013/2014, and 2014/2015) as identified by the National Influenza Surveillance Network. When these are considered, the results of these two approaches provided a better understanding of the impact of the new influenza A(H1N1)pdm09 variant during predominant seasons on influenza A(H1N1)pdm09 virus-associated complications.

## Results

### National Influenza Surveillance Network data on influenza A(H1N1)pdm09 virus during the 2009/2010 to 2015/2016 seasons

**Figure 1:**
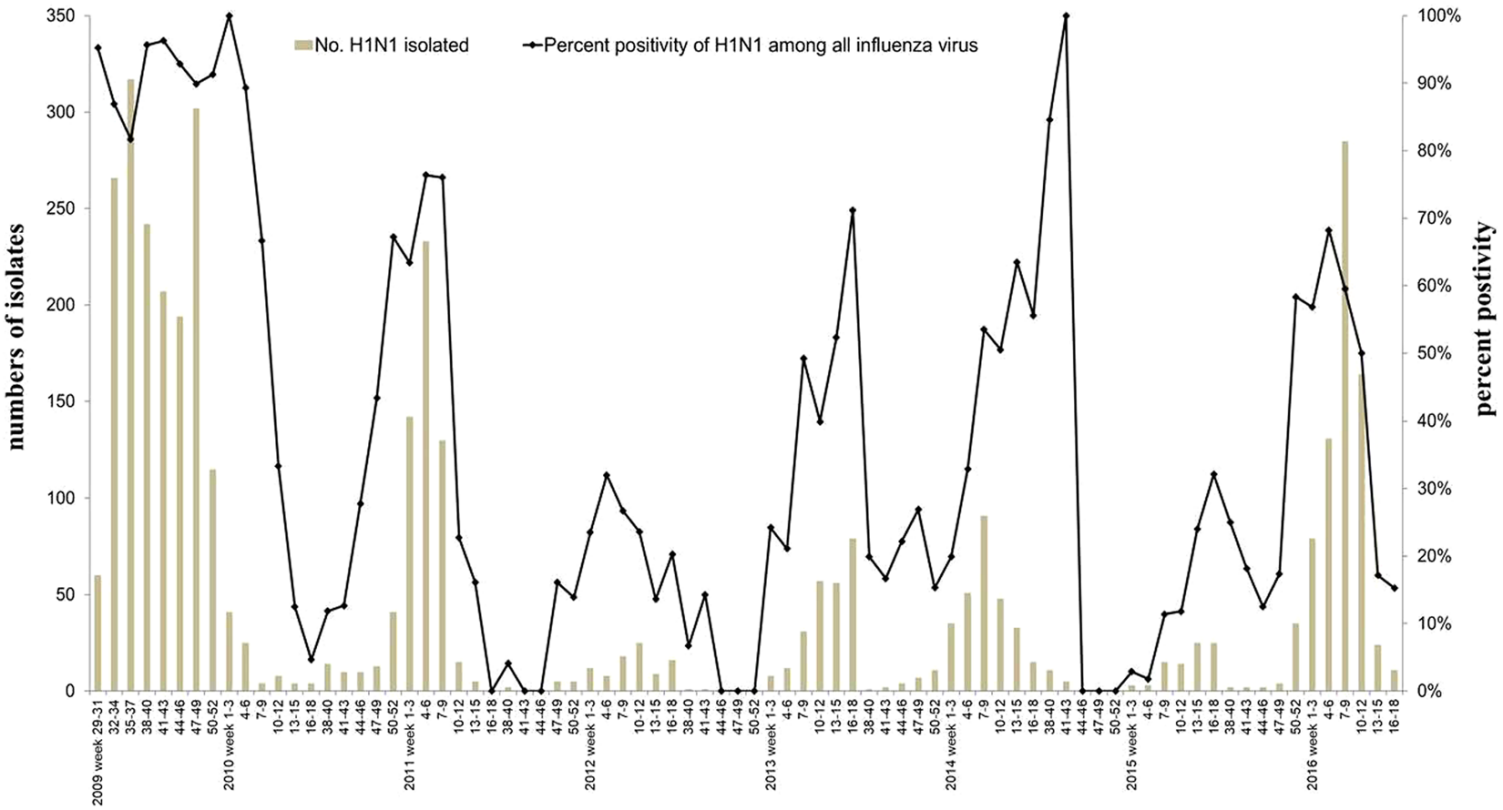
Isolate numbers of influenza A(H1N1)pdm09 virus and percentage of influenza A(H1N1)pdm09 virus among all influenza viruses during 2009/2010 to 2015/2016 seasons. Data for weeks 19–37 were not available.

**Figure 2:**
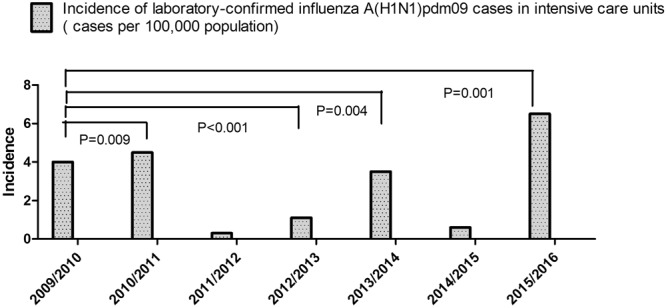
Incidence of laboratory-confirmed influenza A(H1N1)pdm09 cases in intensive care units in the 2009/2010, 2010/2011, 2011/2012, 2012/2013, 2013/2014, 2014/2015, and 2015/2016 seasons (*P* value obtained using Poisson regression for the incidence rate ratio) in Tawian.

Based on the *Taiwan Influenza Express* between July 2009 and May 2016, A(H1N1)pdm09 caused the highest community attack rate (the number of individuals who were infected divided by the number of individuals at risk) in the 2009/2010 season (Fig. 1) compared to other seasons. In addition to 2009/2010, the influenza A(H1N1)pdm09 also predominated in 2010/2011, 2012/2013, 2013/2014, and 2015/2016 (Fig. 1). However, the incidence of laboratory-confirmed influenza A(H1N1)pdm09 cases in intensive care units (ICUs) was highest in 2015/2016 (6.47 cases per 100,000 population), followed by the 2010/2011 season (4.5 cases per 100,000 population); both values were significantly higher than the incidence of 4.0 cases per 100,000 population in 2009/2010 (2015/2016 vs 2009/2010: incidence rate ratio (IRR): 1.6; 95% CI: 1.5–1.8; *P* = 0.001 by Poisson; 2010/2011 vs 2009/2010: IRR: 1.1; 95% CI: 1.03–1.2; *P* = 0.009) (Fig. 2). The incidence of laboratory-confirmed influenza A(H1N1)pdm09 cases in the ICU was 1.1 cases in 2012/2013 and 3.5 cases in 2013/2014 per 100,000 population, a value that was lower than that in 2009/2010 (2012/2013 vs 2009/2010: IRR: 0.27; 95% CI: 0.2–0.3; *P* < 0.001; 2013/2014 vs 2009/2010: IRR: 0.87; 95% CI: 0.79–0.96; *P* = 0.004).

### Phylogenetic analysis of the HA gene

We identified 639 patients with laboratory-confirmed influenza A(H1N1)pdm09 virus infection from the outpatient, inpatient, and emergency departments of Chang Gung Memorial Hosptial (CGMH) in the 2009/2010, 2010/2011, 2012/2013, 2013/2014, and 2015/2016 seasons. This group comprised 248 patients in 2009/2010, 152 patients in 2010/2011, 32 patients in 2012/2013, 81 patients in 2013/2014, and 126 patients in 2015/2016 (Table 1). The percentages of patients included in each department were significantly different between each season (Table 1). A phylogenetic analysis of hemagglutinin (HA) sequences recovered in these epidemics, along with geographically diverse global influenza A(H1N1) pdm09 viral sequences, has revealed that the sequences are members of clades 1, 2, and 8 in 2009/2010; clades 3, 4, 5, and 7 in 2010/2011; clades 7 and 6C in 2012/2013; clade 6B in 2013/2014; and clades 6B, 6B.1, and 6B.2 in 2015/2016 (Fig. S1A). The neuraminidase genes of these viruses were sequencing as well (Fig. S1B). However, no specific amino acid substitutions associated with influenza-related complications was found in each season.

**Table 1:** Patient Numbers and Percentages Included in Each Department During the 2009/2010, 2010/2011, 2012/2013, 2013/2014, and 2015/2016 Seasons

	N, %			*P* value
	Emergency	Outpatient	Inpatient	
2009/2010	15 (6)	51 (20.6)	182 (73.4)	<0.001
2010/2011	14 (9.2)	41 (27)	97 (63.8)	
2012/2013	10 (31.2)	14 (43.8)	8 (25)	
2013/2014	3 (3.7)	25 (30.9)	53 (65.4)	
2015/2016	0 (0)	22 (17.5)	104 (82.5)	

### Demographic and clinical characteristics of patients with influenza A(H1N1)pdm09 virus clade 6B/6B.1/6B.2 and non-clade 6B/6B.1/6B.2 infection

To compare the effects of the clade 6B/6B.1/6B.2 seasons versus non-clades 6B/6B.1/6B.2 seasons, the patients were classified into two groups. The median (interquartile range, IQR) age of patients in the 6B/6B.1/6B.2 season was older than that in the non 6B/6B.1/6B.2 seasons (Table 2). The number of infected individuals aged 50–64 years was higher in 6B/6B.1/6B.2 seasons than that in the non-clade 6B/6B.1/6B.2 seasons (Table 2). The rate of underlying conditions; complications, including pneumonia and acute respiratory distress syndrome (ARDS); ICU admission; respiratory failure with mechanical ventilation; 30-day mortality; and in-hospital mortality in 6B/6B.1/6B.2 seasons were significantly higher than that in non-clade 6B/6B.1/6B.2 seasons (Table 2). A higher rate of patients received antiviral treatment after 48 hours in 6B/6B.1/6B.2 seasons (Table 2). Among the female patients, three were pregnant.

**Table 2:** Demographic and Clinical Characteristics of Patients with A(H1N1)pdm09 Infection in the clade 6B/6B.1/6B.2-predominant seasons and non-clade 6B/6B.1/6B.2-predominant seasons.

Characteristics	Total	Seasons 2009/2010, 2010/2011, 2012/2013 2013/2014, 2015/2016		*P* value
	N = 639 (%)	Non-6B/6B.1/6B.2 N = 432 (%)	6B/6B.1/6B.2 N = 207 (%)	
Gender, male	366 (57.3)	251 (58.1)	115 (55.6)	0. 6
Age, median (IQR), years	12.2 (4–38.1)	10.3 (4.2–29.3)	29.8 (3.1–52.4)	<0.001
Age group				<0.001
≤5	219 (34.3)	146 (33.8)	73 (35.3)	
6–18	151 (23.6)	129 (29.9)	22 (10.6)	
19–49	170 (26.6)	115 (26.6)	55 (26.6)	
50–64	64 (10.0)	26 (6)	38 (18.4)	
≥65	35 (5.5)	16 (3.7)	19 (9.2)	
Onset to presentation, median (IQR), days	2 (1–3)	2 (1–3)	2 (1–4)	0.15
Underlying conditions	140 (21.9)	67 (15.5)	73 (35.3)	<0.001
Chronic respiratory disease	14 (2.2)	7 (1.6)	7 (3.4)	0.16^a^
Cardiovascular disease	73 (11.4)	28 (6.5)	45 (21.7)	<0.001
Neurologic disease	24 (3.8)	12 (2.8)	12 (5.8)	0.08
Chronic liver disease	16 (2.5)	9 (2.1)	7 (3.4)	0.4
Chronic renal disease	21 (3.3)	8 (1.9)	13 (6.3)	0.005
Asthma	19 (3.0)	14 (3.2)	5 (2.4)	0.6
Diabetes mellitus	36 (5.6)	17 (3.9)	19 (9.2)	0.01
Immunosuppression	23 (3.6)	10 (2.3)	13 (6.3)	0.01
Obesity^b^	37/426 (8.7)	21/288 (7.3)	16/138 (11.6)	0.2
Smoking	35 (5.5)	20 (4.6)	15 (7.2)	0.2
Alcoholism	6 (0.9)	3 (0.7)	3 (1.4)	0.4^a^
White blood cell count^c^ (×10^3^/uL)	7.4 (5.2–10.1)	7.3 (5.3–10.1)	7.5 (5.1–10.3)	0.7
Complication	179 (28)	95 (22.0)	84 (40.6)	<0.001
Pneumonia	162 (25.4)	84 (19.4)	78 (37.7)	<0.001
ARDS	41 (6.4)	13 (3.0)	28 (13.5)	<0.001
Seizure	5 (0.8)	3 (0.7)	2 (1.0)	0.7^a^
Encephalitis	8 (1.3)	6 (1.4)	2 (1.0)	1.0^a^
Meningitis	1 (0.2)	0 (0.0)	1 (0.5)	0.3^a^
Stroke	1 (0.2)	0 (0.0)	0 (0.0)	0.3^a^
Myocarditis	5 (0.8)	2 (0.5)	3 (1.4)	0.3^a^
Myocardial infarction	1 (0.2)	0 (0.0)	1 (0.5)	0.3^a^
Received antiviral therapy	417 (65.3)	267 (61.8)	150 (72.5)	0.01
Within 48 hours	247 (38.7)	169 (39.1)	78 (37.7)	
After 48 hours	170 (26.6)	98 (22.7)	72 (34.8)	
Interval from symptom onset to antiviral therapy, median (IQR), days	2 (1–4)	2 (1–3)	2 (1–4)	0.04
Hospital admission for any reason	453 (70.9)	296 (68.5)	157 (75.8)	0.06
ICU admission	79 (12.4)	33 (7.6)	46 (22.2)	<0.001
Mechanical ventilation	72 (11.3)	30 (6.9)	42 (20.3)	<0.001
30-day mortality	32 (5.0)	15 (3.5)	17 (8.2)	0.01
In-hospital mortality	37 (5.8)	16 (3.7)	21 (10.1)	0.001

^a^Fisher’s exact test.

^b^Case numbers with available BMI.

^c^WBC data were available in 476 patients.

### Demographic and clinical characteristics of patients with complications after influenza A(H1N1)pdm09 virus clade 6B/6B.1/6B.2 and non-clade 6B/6B.1/6B.2 infection

Table 3 showed the baseline characteristics of patients with influenza complications in 6B/6B.1/6B.2 seasons and non-clade 6B/6B.1/6B.2 seasons. The number of infected individuals aged ≥50 years was higher in 6B/6B.1/6B.2 seasons than in non-clade 6B/6B.1/6B.2 seasons (Table 3). The rate of underlying conditions; complications, such as ARDS; ICU admission; and respiratory failure with mechanical ventilation in 6B/6B.1/6B.2 seasons was significantly higher than that in non-clade 6B/6B.1/6B.2 seasons (Table 3).

**Table 3:** Demographic and Clinical Characteristics of Patients with Influenza A(H1N1)pdm09-associated complication in the clade 6B/6B.1/6B.2-predominant seasons and non-clade 6B/6B.1/6B.2-predominant seasons.

Characteristics	Total	Seasons 2009/2010, 2010/2011, 2012/2013 2013/2014, 2015/2016		*P* value
	N = 179 (%)	Non-6B/6B.1/6B.2 N = 95 (%)	6B/6B.1/6B.2 N = 84 (%)	
Gender, male	96 (53.9)	47 (49.5)	49 (58.3)	0.3
Age, median (IQR), years	33.8 (6.5–56.1)	12.8 (5.9–45)	48.6 (13.7–61.1)	<0.001
Age group				<0.001
≤5	38 (21.3)	25 (26.3)	13 (15.5)	
6–18	41 (23)	30 (31.6)	12 (14.3)	
19–49	39 (21.9)	21 (22.1)	18 (21.4)	
50–64	35 (19.7)	11 (11.6)	24 (28.6)	
≥65	25 (14)	8 (8.4)	17 (20.2)	
Onset to presentation, median (IQR), days	2 (1–2)	3 (1–3)	2 (1–2)	0.3
Underlying conditions	82 (46.1)	33 (34.7)	49 (58.3)	0.002
Chronic respiratory disease	7 (3.9)	3 (3.2)	4 (4.8)	0.7^a^
Cardiovascular disease	51 (28.7)	17 (17.9)	34 (40.5)	0.001
Neurologic disease	18 (10.1)	9 (9.5)	9 (10.7)	0.8
Chronic liver disease	11 (6.2)	5 (5.3)	6 (7.1)	0.8
Chronic renal disease	17 (9.6)	6 (6.3)	11 (13.1)	0.1
Asthma	4 (2.2)	3 (3.2)	1 (1.2)	0.6^a^
Diabetes mellitus	28 (15.7)	10 (10.5)	18 (21.4)	0.06
Immunosuppression	17 (9.6)	7 (7.4)	10 (12)	0.3
Obesity^b^	29/151 (19.2)	16/87 (18.4)	13/64 (20.3)	0.8
Smoking	26 (14.6)	12 (12.6)	14 (16.7)	0.5
Alcoholism	5 (2.8)	2 (2.1)	3 (3.6)	0.7^a^
White blood cell count (×10^3^/uL)	7.2 (4.75–10.4)	7.1 (4.8–10.4)	8.15 (4.65–10.4)	0.3
Complication				
Pneumonia	162 (90.5)	84 (88.4)	78 (92.9)	0.4
ARDS	41 (23)	13 (13.7)	28 (33.3)	0.002
Seizure	5 (2.8)	3 (3.2)	2 (2.4)	1.0^a^
Encephalitis	8 (4.5)	6 (6.3)	2 (2.4)	0.3^a^
Meningitis	1 (0.6)	0 (0)	1 (1.2)	0.5^a^
Stroke	1 (0.6)	0 (0)	1 (1.2)	0.5^a^
Myocarditis	5 (2.8)	2 (2.1)	3 (3.6)	0.7^a^
Myocardial infarction	1 (0.6)	0 (0)	1 (1.2)	0.5^a^
Received antiviral therapy	156 (87.6)	82 (86.3)	75 (89.3)	0.8
Within 48 hours	72 (40.4)	37 (38.9)	35 (41.7)	
After 48 hours	84 (47.2)	45 (47.4)	40 (47.6)	
Interval from symptom onset to antiviral therapy, median (IQR), days	3 (1–5)	3 (1–5)	3 (1–5)	0.7
ICU admission	79 (44.4)	33 (34.7)	46 (54.8)	0.01
Mechanical ventilation	72 (40.4)	30 (31.6)	42 (50)	0.01
30-day mortality	32 (18)	15 (15.8)	17 (20.2)	0.6
In-hospital mortality	37 (20.8)	16 (16.8)	21 (25)	0.2

^a^Fisher’s exact test.

^b^Case numbers with available BMI.

### Risk factors of complications in patients with influenza A(H1N1)pdm09 virus infection

The results of the logistic regression analysis on the risk factors associated with influenza A(H1N1)pdm09-related complications and pneumonia are shown in Table 4, and respiratory failure with mechanical ventilation and ARDS are also presented in Table 5. In the univariate analysis, 6B/6B.1/6B.2 season, age (50–64 years), onset to presentation, underlying conditions, obesity, smoking, alcoholism, and antiviral therapy were significant risk factors of complications, pneumonia, mechanical ventilation, and ARDS (Tables 4 and 5). In the multivariate logistic regression analysis, 6B/6B.1/6B.2 season, age (50–64 years and ≥65 years), underlying conditions, and antiviral therapy were significant independent risk factors of complications, pneumonia, and mechanical ventilation (Tables 4 and 5). Only 6B/6B.1/6B.2 season and obesity were considered as significant independent risk factors of ARDS (Table 5). The effect of 6B/6B.1/6B.2 season on the total number of influenza-related complications was not significant in children aged ≤5 years. However, it was significantly stronger among individuals aged ≥6 years (Table S1).

**Table 4:** Logistic Regression Analysis of the Risk Factors Associated with Complications and Pneumonia among Patients with Influenza A(H1N1)pdm09 Virus Infection.

Variables	Total number of complications				Pneumonia			
	Univariate analysis		Multivariate analysis		Univariate analysis		Multivariate analysis	
	OR (95% CI)	*P* value	OR (95% CI)	*P* value	OR (95% CI)	*P* value	OR (95% CI)	*P* value
Gender, male	0.8 (0.6–1.2)	0.3			0.9 (0.6–1.3)	0.5		
6B/6B.1/6B.2 season	2.4 (1.7–3.4)	<0.001	1.6 (1.0–2.8)	0.05	2.5 (1.7–3.6)	<0.001	1.78 (1.04–3.04)	0.03
Age group		<0.001		0.01		<0.001		0.003
≤5	Reference		Reference		Reference		Reference	
6–18	1.8 (1.1–2.9)	0.03	2.1 (1.2–3.9)	0.01	1.8 (1.1–3.1)	0.03	2.6 (1.4–4.9)	0.003
19–49	1.4 (0.9–2.3	0.17	2.04 (0.9–4.4)	0.07	1.7 (1.0–2.9)	0.05	2.5 (1.1–5.4)	0.03
50–64	5.7 (3.1–10.5)	<0.001	3.7 (1.3–10.3)	0.01	6.9 (3.7–12.8)	<0.001	4.04 (1.5–11.2)	0.007
≥65	11.9 (5.3–26.8)	<0.001	4.2 (1.2–14)	0.02	15.2 (6.6–34.6)	<0.001	5.7 (1.7–19.6)	0.005
Onset to presentation	1.1 (1.0–1.2)	0.008	1.05 (0.97–1.13)	0.25	1.1 (1.04–1.2)	0.003	1.05 (0.97–1.13)	0.3
Underlying conditions	5.9 (3.97–8.9)	<0.001	2.7 (1.45–5.04)	0.002	6.5 (4.3–9.8)	<0.001	2.9 (1.6–5.5)	0.001
Obesity	6.6 (3.1–13.9)	<0.001	2.53 (1.03–6.2)	0.04	5.8 (2.8–11.8)	<0.001	2.23 (0.9–5.4)	0.08
Smoking	8.6 (3.9–18.7)	<0.001	2.7 (0.8–9.1)	0.1	8.5 (4.0–18.2)	<0.001	2.15 (0.7–6.9)	0.2
Alcoholism	13.3 (1.5–114.6)	0.02	1.7 (0.1–19.6)	0.7	15.2 (1.8–130.7)	0.01	2.5 (0.3–0.1)	0.47
Antiviral therapy		<0.001		<0.001		<0.001		<0.001
No antiviral therapy	Reference		Reference		Reference		Reference	
Within 48 hours	3.7 (2.2–6.3)	<0.001	2.7 (1.4–5.3)	0.003	3.2 (1.9–5.6)	<0.001	1.9 (0.93–3.8)	0.08
After 48 hours	8.9 (5.2–15.1)	<0.001	3.7 (1.9–7.1)	<0.001	9.4 (5.4–16.3)	<0.001	3.7 (1.9–7.2)	<0.001

**Table 5:** Logistic Regression Analysis of the Risk Factors Associated with the Mechanical Complication and Acute Respiratory Distress Syndrome (ARDS) among Patients with Influenza A(H1N1)pdm09 Virus Infection.

Variables	Mechanical Ventilation				ARDS			
	Univariate analysis		Multivariate analysis		Univariate analysis		Multivariate analysis	
	OR (95% CI)	*P* value	OR (95% CI)	*P* value	OR (95% CI)	*P* value	OR (95% CI)	*P* value
Gender, male	1.1 (0.7–2.0)	0.5			1.5 (0.8–2.9)	0.3		
6B/6B.1/6B.2 season	3.4 (2.1–5.6)	<0.001	2.6 (1.2–5.6)	0.02	5.0 (2.6–9.96)	<0.001	5.5 (1.9–15.9)	0.002
Age group		<0.001		0.006		0.02		0.9
≤5	Reference		Reference		—^a^	—^a^	—^a^	—^a^
6–18	0.7 (0.2–2.4)	0.6	0.8 (0.2–3.4)	0.76	—^a^	—^a^	—^a^	—^a^
19–49	3.7 (1.6–8.6)	0.002	4.0 (1.3–12.6)	0.02	Reference		Reference	
50–64	12.9 (5.4–31)	<0.001	3.9 (1.1–13.8)	0.04	2.8 (1.3–5.9)	0.007	0.8 (0.2–2.6)	0.7
≥65	27.9 (10.6–73.6)	<0.001	9.6 (2.3–39.6)	0.002	2.1 (0.8–5.5)	0.1	0.7 (0.16–2.9)	0.6
Onset to presentation	1.1 (1.02–1.15)	0.008	1.04 (0.96–1.13)	0.3	1.09 (1.03–1.16)	0.006	1.1 (0.98–1.25)	0.1
Underlying conditions	14.2 (8.1–24.9)	<0.001	5.5 (2.34–13.1)	<0.001	10.6 (5.2–21.4)	<0.001	2.1 (0.6–6.7)	0.2
Obesity	7.1 (3.6–14.3)	<0.001	2.4 (0.88–6.6)	0.08	11.7 (5.4–25.3)	<0.001	3.0 (1.04–8.7)	0.04
Smoking	10.8 (5.3–22.1)	<0.001	2.24 (0.7–7.3)	0.2	16.7 (7.7–36.2)	<0.001	2.9 (0.9–9.7)	0.09
Alcoholism	42.2 (4.9–367)	0.001	8.2 (0.6–109.1)	0.1	32.2 (5.7–181.6)	<0.001	3.8 (0.4–39.3)	0.27
Antiviral therapy		<0.001		0.03		<0.001		0.05
No antiviral therapy	Reference		Reference		Reference		Reference	
Within 48 hours	8.2 (2.4–27.6)	0.001	3.4 (0.8–14.8)	0.1	12.3 (1.6–94.6)	0.02	5.2 (0.2–124.6)	0.3
After 48 hours	25.5 (7.8–83.8)	<0.001	8.5 (2.1–33.9)	0.002	41.7 (5.6–310.5)	<0.001	13.4 (0.7–268.9)	0.09

^a^Data cannot be calculated due to absence of ARDS in the age group.

### Risk factors of mortality in patients with influenza A(H1N1)pdm09

Among the hospitalized patients with laboratory-confirmed influenza A(H1N1)pdm09 infection, male patients and those with underlying conditions were significantly at risk for 30-day mortality (overall death within the first 30 days after hospital admission) and all-cause in-hospital mortality (overall death during hospital admission) as assessed using the multivariable Cox proportional hazard model (Table 6). The same analysis showed that season was not associated with an increased risk for 30-day and all-cause in-hospital mortality (Table 6).

**Table 6:** Hazard Ratios for Influenza-related 30-days Mortality and In-hospital Mortality among 453 Hospitalized Patients.

Variables	30-day mortality				In-hospital mortality			
	Univariate analysis		Multivariate analysis		Univariate analysis		Multivariate analysis	
	Hazard Ratio (95% CI)	*P* value	Hazard Ratio (95% CI)	*P* value	Hazard Ratio (95% CI)	*P* value	Hazard Ratio (95% CI)	*P* value
Gender, male	3.5 (1.4–8.6)	0.006	3.17 (1.3–7.9)	0.01	2.8 (1.3–5.9)	0.009	2.96 (1.3–6.8)	0.01
6B/6B.1/6B.2 season	1.4 (0.68–2.8)	0.37			1.5 (0.8–2.9)	0.2		
Age group		0.002		0.14		0.003		0.2
≤5	Reference		Reference		Reference		Reference	
6–18	3.1 (0.5–18.6)	0.2	3.4 (0.6–20.2)	0.19	3.2 (0.5–19.2)	0.2	3.5 (0.6–21)	0.17
19–49	9.4 (1.98–44.8)	0.005	3.6 (0.7–18.3)	0.13	10.4 (2.2–48.1)	0.003	4.7 (0.96–22.8)	0.06
50–64	6.8 (1.4–34.1)	0.02	2.09 (0.4–11.1)	0.4	7.7 (1.6–37.2)	0.01	2.7 (0.5–14.2)	0.2
≥65	18.1 (3.9–84.5)	<0.001	5.6 (1.1–27.7)	0.04	16.6 (3.5–77.6)	<0.001	5.5 (1.1–27.3)	0.04
Onset to presentation	0.99 (0.9–1.1)	0.79			0.98 (0.9–1.1)	0.7		
Underlying conditions	10.3 (3.5–30.5)	<0.001	7.2 (2.2–23.7)	0.001	8.5 (3.2–22.7)	<0.001	6.9 (2.4–20.2)	<0.001
Obesity	1.0 (0.36–2.8)	1			0.8 (0.3–2.04)	0.6		
Smoking	1.7 (0.74–3.9)	0.2			1.9 (0.9–4.1)	0.09	0.7 (0.3–1.7)	0.5
Alcoholism	4.5 (1.3–15.6)	0.02	2.7 (0.74–10.1)	0.13	4.5 (1.3–15.6)	0.02	2.8 (0.7–10.4)	0.1
Antiviral therapy	—^a^	—^a^	—^a^	—^a^	—^a^	—^a^	—^a^	—^a^

^a^Data cannot be calculated because none of the patients who did not receive antiviral therapy died.

## Discussion

The present study assessed the clinical characteristics and complications of patients with influenza A(H1N1)pdm09 virus during the five A(H1N1)pdm09-predominant seasons over 7 years, including the 2009/2010, 2010/2011, 2012/2013, 2013/2014, and 2015/2016 seasons. Consistent with previous reports^12,13^, we found that age, underlying disease, and obesity were significant risk factors for A(H1N1)pdm09-related complications. Early antiviral treatment that reduces influenza-related complications, including lower respiratory tract infection, was evaluated^14^. In the present study, the association between antiviral treatment and the occurrence of complications should be attributed to the antiviral treatment frequently used in patients with influenza-related complications. Furthermore, we observed that the severity of influenza differed significantly, especially in individuals aged ≥6 years, during the 6B/6B.1/6B.2-predominant and 6B/6B.1/6B.2-non-predominant seasons.

After the 2009 pandemic, when the 2009 H1N1 virus became a regularly circulating seasonal influenza strain, the WHO recommended the use of the A/California/7/2009 (H1N1)pdm09-like virus as a vaccine strain. In the face of a cumulatively established population immunity, the A(H1N1)pdm09 virus would need to exhibit antigenic changes to avoid suppression by the host immune system. To date, eight genetic virus groups have been identified^15^. In 2013/2014, clade 6B appeared, achieving general dominance over other influenza viruses^4^. During the 2013/2014 season, an unusually high hospitalization rate in adults aged 50–64 years was observed in the United States and Mexico^3,16^. The increased morbidity in middle-aged adults during the 2013/2014 season had been attributed to the low vaccination rate in this age group^16^. However, that hypothesis cannot explain the unusual number of severe cases because the vaccination rate had already been low during the previous years^16,17^. An interesting study has shown that up to 42% of middle-aged adults born between 1965 and 1979, who had been exposed to seasonal H1N1 viruses circulating in 1977, had reduced serologic reactivity with the 2013/2014 A(H1N1)pdm09; notably, the 2013/2014 virus harbors the distinctive K163Q HA antigenic mutation^18^. In the cohort of individuals born between 1965 and 1979, HA-specific antibodies with activity against A(H1N1)pdm09 must have been produced and shaped by exposure to prior-season H1N1 viruses (the so called “original antigenic sin”)^19^. Nonetheless, the HA-specific antibodies in this cohort failed to recognize the 2013/2014 A(H1N1)pdm09 genetic variant^18^. This explains the high susceptibility of the members of this middle-aged cohort to the 2013/2014 A(H1N1)pdm09 viruse. Influenza A(H1N1)pdm09 viruses circulating globally in the 2015/2016 season are members of the phylogenetic clade 6B.1/6B.2, which prevailed in Eastern Europe and Western Asia, resulting in widespread influenza activity and severity^20–22^. In Russia and Israel, 6B.1/6B.2 caused increases in influenza-associated morbidity and mortality^5,21^. The present study showed that individuals in the 6B/6B.1/6B.2 seasons compared with non-clade 6B/6B.1/6B.2 seasons were 1.6-, 1.78-, 2.6-, and 5.5-fold at greater risk for any complications, pneumonia, mechanical ventilation, and the development of ARDS, respectively. Clinically, influenza A(H1N1)pdm09 virus is associated with a greater risk of lower respiratory tract infection^23,24^. The 2013/2014 clade 6B and 2015/2016 A(H1N1)pdm09 clade 6B.1/6B.2 variants are more likely to cause severe pneumonia and acute life-threatening respiratory failure in a range of individuals.

Research has shown that clade-6B A(H1N1)pdm09 viruses, including those in subclades 6B.1 and 6B.2, were antigenically distinguishable from the A/California/7/2009 vaccine virus when tested with the human post-vaccination sera^25^. Compared to the A/California/7/2009 vaccine virus, viruses of clade 6B harbor D97N, K163Q, S185T, K283E, and A256T substitutions in HA1. Viruses of subclade 6B.1 harbor further amino acid substitutions S84N, S162N, and I216T and 6B.2 and carry amino acid substitutions V152T and V173I^20^. More extensive studies must be conducted to identify the potential antigenic differences between clade 6B and subclade 6B.1/6B.2; such studies are expected to improve our understanding of how A(H1N1)pdm09 evolved (and continues to evolve) and how it affects and interacts with the human immune system. In the 2017/2018 season, the WHO has selected a new vaccine virus, which is the A/Michigan/45/2015 (H1N1)pdm09-like virus (a member of the 6B.1 subclade), as the influenza vaccine virus component for the northern hemisphere.

The National Influenza Surveillance Network coordinated by the Taiwan CDC was established more than 10 years ago. Policies favoring government funding for vaccines and antiviral agents have been consistent during the subsequent intervals. Between 2009 and 2015, government-funded vaccines have been administered primarily to those aged 6 months to 12 years, elderly individuals aged ≥65 years, healthcare workers, and individuals with underlying diseases. Individuals aged 13–64 years were not included in the government-funded vaccination program. Elementary school children aged 7–12 years had the highest influenza vaccination rate, with coverage reaching 60–70% annually^11^.

The present study is limited by its observational nature and the incorporation of a retrospective investigation. A potential bias may exist due to the exclusion of all cases with A(H1N1)pdm09 infection for 7 years. Nonetheless, no change was observed in terms of admission or management procedures during these outbreaks. The surveillance and reporting system in Taiwan has long been established. Taken together, the increased frequency of complications in 2013/2014 and 2015/2016 is unlikely due to detection bias. In addition, the major drawback of this study was the lack of documentation about the history of influenza vaccination in the records used to generate this study. However, a study by Taiwan CDC has reported that 95% of patients with complications in the 2015/2016 season had not received the influenza vaccine^26^. The vaccine coverage rate in non-elderly adults and elderly individuals would have been low during each of the outbreaks, particularly during the first wave, given that no A(H1N1)pdm09 vaccine was available in 2009/2010. Thus, the increased severity of influenza during the 2013/2014 and 2015/2016 seasons is unlikely to reflect a decreased rate of vaccination. The study has shown that Taiwan experienced the greatest burden of influenza-related complications due to A(H1N1)pdm09 clades 6B/6B.1/6B.2 in the sixth year of its circulation. The reasons for the increased impact of influenza-related complications remain uncertain. Aspects related to the antigenic change of A(H1N1)pdm09 virus, immune response of the host, and environmental factors required further investigation. This report shows the importance of influenza disease surveillance and requires that the influenza A(H1N1)pdm09 virus should always be considered.

## Methods

### National influenza surveillance network

The network consists of eight regional commissioned laboratories located in the northern (n = 3), central (n = 2), southern (n = 2), and eastern (n = 1) parts of Taiwan. These laboratories have steadily collected more than 8000 respiratory specimens for surveillance per year, including more than 1000 influenza virus specimens annually, all of which are sent to the Taiwan CDC for the monitoring of influenza viral activity. The *Taiwan Influenza Express*, a weekly online influenza surveillance report, has been published by the Taiwan CDC from July to May of each year since 2005 (http://www.cdc.gov.tw/english/submenu.aspx?treeid = 00ED75D6C887BB27&nowtreeid = 8F1E239D0FD8877A)^10,27^. This report includes the total number of respiratory specimens; isolate number of influenza A(H1N1), influenza A(H3N2), and influenza B; and case number of laboratory-confirmed influenza cases in intensive care units (ICUs), a class of events that is considered a category 4 nationally notifiable disease. However, data on weeks 19–37 are not available annually. A confirmed case involved a patient who had acute influenza-like illness (temperature ≥ 38 °C with either cough or sore throat) and nasopharyngeal/throat or bronchoalveolar lavage samples harboring influenza A(H1N1)pdm09 virus as detected using real-time (RT) reverse-transcription polymerase chain reaction (PCR) assay or via viral culture^11,28^. For the purposes of the present study, each season was defined as extending from July of the same year to May of the following year. The annual population figures provided by the Department of Household Registration Affairs of the Interior Ministry were used for the calculation of the incidence of laboratory-confirmed influenza A(H1N1)pdm09 cases in the ICU.

### A retrospective cohort study

Based on the data obtained from the National Influenza Surveillance Network, 2009/2010, 2010/2011, 2012/2013, 2013/2014, and 2015/2016 were considered as A(H1N1)pdm09-predominant seasons. Therefore, we conducted a retrospective observational cohort study for the 2009/2010, 2010/2011, 2012/2013, 2013/2014, and 2015/2016 seasons. This study was performed at the Chang Gung Memorial Hospital (CGMH), a 4000-bed, university-affiliated teaching hospital that is located in northern Taiwan and provides both primary and tertiary care. In addition, CGMH is one of the 8 regional commissioned laboratories of the Taiwan CDC. Patients who had acute influenza-like illness (temperature ≥ 38 °C with either cough or sore throat) and had influenza A(H1N1)pdm09 virus as detected using RT-PCR assay or via viral culture using respiratory specimens were included in the study. Patients whose data are not available were excluded. The institutional review board of CGMHT approved the study, and it was carried out in accordance with the relevant guidelines and regulations. Informed consent was waived due to the study’s retrospective nature. All medical records of the enrolled patients were reviewed. Demographic characteristics, underlying medical conditions, clinical course, antiviral treatment (oseltamivir or zanamivir), mechanical ventilation, admission to an ICU, and death were recorded using a structured questionnaire. Body mass index (BMI), a measure of obesity, was calculated for patients whose height and weight data were available. Obesity was defined as follows: 1) body weight ≥ 95th percentile in children < 2 years of age; 2) BMI ≥ 25 kg/m^2^ in patients aged between 2 and 18 years; and 3) BMI > 28 kg/m^2^ (Chinese criteria) in patients > 18 years^29^. Medical conditions associated with a high risk for influenza complications were defined based on those listed by the United States Advisory Committee on Immunization Practices^30^. Patients with confirmed pneumonia on radiography, acute respiratory distress syndrome (ARDS), acute onset of cardiovascular, neurologic condition, respiratory failure with mechanical ventilation; those who were admitted in the ICU; and those who died were considered to have influenza-related complications. Pneumonia on radiography was diagnosed based on the presence of a consolidation, infiltrate, or opacity^31^. ARDS was defined according to the standard criteria^32^. The primary study outcome was the occurrence of (any) influenza-related complications. The secondary study outcomes were pneumonia, mechanical ventilation, ARDS, 30-day mortality, and in-hospital mortality.

### Genetic characterization of the virus

A total of 82 isolated influenza A(H1N1)pdm09 virus were randomly selected for the analysis of viral hemagglutinin (HA) and neuraminidase (NA) genes across the five seasons. The RNA was extracted using the QIAamp Viral RNA mini kit (Qigen, Germany) according to the manufacturer’s instructions. RT-PCR and primer pairs used for sequencing HA and NA genes were performed, as previously described^33^. Sanger sequencing of the viral HA and NA genes was performed to establish clade designation and to detect differences in amino acid^33^. The obtained amplicons were assembled into a full-length 1,701-bp span for HA and 1410-bp for NA using DNASTAR Lasergene (DNAStar, Madison, WI). Newly reported sequences in this study were deposited at the GenBank database under the accession numbers shown in Fig. S1 for HA and NA genes. The evolution history was inferred by the maximum likelihood method based on the Hasegawa–Kishino–Yano model^34^. The percentages of replicate trees (1,000 replicates) are shown next to the branches in which the associated taxa clustered together in the bootstrap test. Phylogenetic analysis in this study was conducted using MEGA7^35^.

### Statistical analysis

Continuous variables were presented as medians and interquartile ranges (IQRs); categorical variables were presented as numbers and percentages. All analyses were performed using the Statistical Package for the Social Sciences software package version 22.0 (SPSS Inc., Chicago, IL, the USA). The incidence rate ratio (IRR) was generated using Poisson regression with 95% confidence intervals to compare the rates of laboratory-confirmed influenza A(H1N1)pdm09 cases in the ICU per 100,000 populations across different seasons; 95% confidence intervals for which the upper and lower bounds did not include 1 were considered as statistically significant. Differences in categorical variables were compared using the chi-square test or a Fisher’s exact test. Continuous variables were compared using the Kruskal–Wallis one-way analysis of variance test. Multivariate logistic regression analysis and multivariate Cox proportional hazards model were used for outcome analysis. The variables included sex, season, age group, onset to presentation, underlying condition, obesity, smoking, alcoholism, and antiviral therapy. Variables with a *P* value < 0.1 in the univariate analysis were included in the multivariate model. The Hosmer–Lemeshow goodness-of-fit test was performed to assess the overall fit of the model. All statistical operations were two-tailed. *P* values ≤ 0.05 were considered statistically significant.

## Electronic supplementary material

Supplementary Dataset 1
