# Supplementary material for: Clinical characteristics of patients with laboratory-confirmed influenza A(H1N1)pdm09 during the 2013/2014 and 2015/2016 clade 6B/6B.1/6B.2-predominant outbreaks
Source: Sci Rep. 2018 Oct 23;8:15636. doi: 10.1038/s41598-018-34077-4 (PMC6199313; doi:10.1038/s41598-018-34077-4)
Supplement: Supplementary file 1 — Supplementary Dataset 1 [file 41598_2018_34077_MOESM1_ESM.docx]

**Clinical characteristics of laboratory-confirmed influenza A(H1N1)pdm09 patients during the 2013/2014 and 2015/2016 clades 6B/6B.1/6B.2 predominant outbreaks**

**Yu-Chia Hsieh^1,＊^, Kuo-Chien Tsao^2,3,4^, Ching-Tai Huang^5^, Kuang-Yi Chang^6,7^, , Yhu-Chering Huang^1^, Yu-Nong Gong^3^**


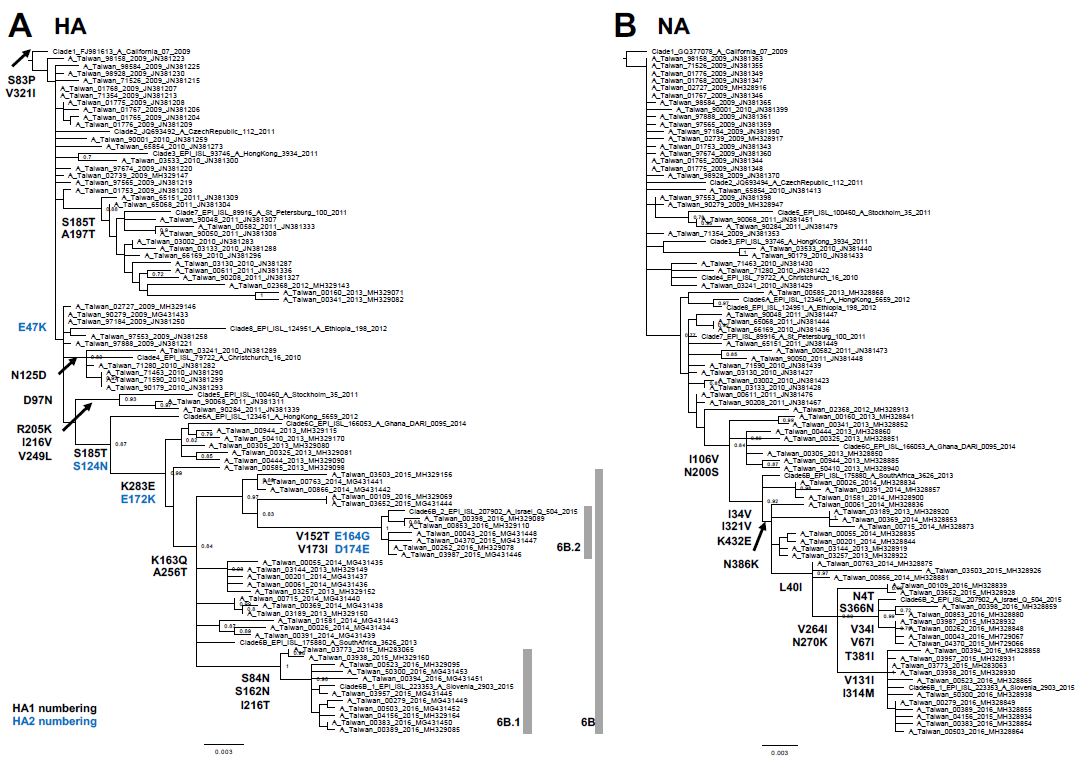


**Supplementary Figure S1.** HA and NA gene phylogeny of influenza A(H1N1)pdm09 viruses detected in the 2009/2010 (n=20; HA accession number: JN381203-4, 381206-9, 381213,381215, 381219-381221, 381223,381225, 381230,381250, 381258-9, MH329146-7, MG431433; NA accession number:JN381343-4, 381346, 381347-9, 381353, 381359-61, 381363, 381365,381370, 381390, 381398-9, MH328942,328947,328916-7), 2010/2011 (n=20; HA: JN381273, 381282-3, 381287-90, 381293, 381296, 381299-300, 381304, 381307-9, 381311,381327, 381333,381336, 381339; NA:JN381413, 381422-3, 381427-30, 381433,381436,381439-40,381444,381447-9,381451,381467,381473,381476,381479), 2012/2013 (n=9; HA: MH329143, 329071, 329080-2, 329090, 329098, 329115, 329170 ; NA: MH328913,328841,328850-52,328860, 328868,328940,328885), 2013/2014 (n=13; HA: MH329149-50, MH329152, MG431434-43; NA:328919-20,328922,328834-6,328844,328853,328857, 328873, 328875, 328881,328900 ), and 2015/2016 seasons (n=20; HA: MG431444-431453, MH283065, 329156, 329069, 329078, 329085, 329089,329095, 329110, 329160,329164; NA:MH328926,328928,283063, 328930-32, 328934, 328938, 328839, 328848-9,328854-5, 328858-9, 328864-5, 328880,729066-7), grouped by 12 reference strains in the maximum likelihood tree. Bootstrap values of 1,000 replicates with greater than 70 are shown. Amino acid changes to each clade are indicated by their HA1 and HA2 numberings. A scale bar represents 0.002 nucleotide difference.

**Supplementary Table S1.** Logistic Regression Analysis of Risk Factor Associated Complications among Patients aged ≥ 6 year with Influenza A(H1N1)pdm09 Virus Infection

| Variable | Total complication | | | |
| --- | --- | --- | --- | --- |
|  | Univariate | | Multivariate | |
|  | OR (95% CI) | *P* | OR (95% CI) | *P* |
| Sex, male | 0.9(0.6-1.4) | 0.8 |  |  |
| 6B/6B.1/6B.2 season | 3.5(2.3-5.4) | <0.001 | 3.4 (1.6-7.1) | 0.002 |
| Age Group |  | <0.001 |  | 0.7 |
| 6 – 18 | Reference |  | Reference |  |
| 19 – 49 | 0.8(0.5-1.3) | 0.3 | 0.65(0.27-1.6) | 0.3 |
| 50 – 64 | 3.1(1.7-5.8) | <0.001 | 0.95(0.26-3.4) | 0.9 |
| ≥ 65 | 6.5(2.9-14.7) | <0.001 | 1.2 (0.3-4.7) | 0.8 |
| Onset to presentation | 1.2(1.1-1.3) | 0.001 | 1.1(0.96-1. 3) | 0.1 |
| Underlying conditions | 6.6(4.1-10.5) | <0.001 | 3.5(1. 5-8.2) | 0.005 |
| Obesity | 3.4(1.6-7.3) | 0.002 | 3.0 (1.1-8.3) | 0.03 |
| Smoking | 10.3(4.1-25.7) | <0.001 | 7.1(1.1-44.7) | 0.04 |
| Alcoholism | 13.3(1.5-114.6) | 0.04 | 0.8(0.05-13.8) | 0.9 |
| Antiviral therapy |  | <0.001 |  | <0.001 |
| No antiviral therapy | Reference |  | Reference |  |
| Within 48 hours | 6.3(2.8-14.3) | <0.001 | 6.7(1.9-23) | 0.002 |
| After 48 hours | 23.3(9.9-54.5) | <0.001 | 12.7(3.7-44.4) | <0.001 |
